# Comparing healthcare utilization among health survey respondents with the total population – are respondents representative?

**DOI:** 10.1186/s12913-016-1745-3

**Published:** 2016-09-22

**Authors:** Janne Agerholm, Daniel Bruce, Bo Burström

**Affiliations:** 1 Department of Public Health Sciences, Karolinska Institutet, 171 77 Stockholm, Sweden; 2 Centre for Epidemiology and Community Medicine, Stockholm County Council, Stockholm, Sweden

**Keywords:** Non-response, Bias, Health survey, Healthcare utilization, Socioeconomic differences, Equity

## Abstract

**Background:**

Surveys are often used for analysis of health status and healthcare utilization in different socioeconomic groups. However, differential non-response rates may bias results. The aim of this study was to compare register data on outpatient healthcare utilization among respondents to a health survey to that of the total population and to investigate whether socioeconomic differences in outpatient healthcare utilization differ between survey respondents and the total population.

**Method:**

Data from the Stockholm Public Health Survey 2010 (*n* = 30,767 aged 18 + years) were linked to register data on outpatient healthcare utilization in order to investigate differentials by socioeconomic groups, country of birth and residential areas among respondents, using logistic regression and negative binomial regression. These results were compared to analyses of register data on outpatient healthcare utilization for the total population (*n* = 1.6 million aged 18 + years) of Stockholm County.

**Results:**

Outpatient healthcare utilization was generally higher among survey respondents than in the total population, especially among men. The proportion of individuals having made at least one visit was significantly higher among survey respondents than in the total population but the differences were smaller regarding the average number of visits. Socioeconomic differences in outpatient healthcare utilization between subgroups were largely similar among survey respondents and in the total population. However, individuals born outside Sweden responding to the survey had significantly higher outpatient healthcare utilisation than individuals born outside Sweden in the total population.

**Conclusion:**

Compared to the total population, a greater proportion of survey respondents had made at least one outpatient visit to the doctor. However, the mean number of registered visits did not differ significantly between survey respondents and the total population. Hence, depending on the outcome measure used survey-based estimates may result in slightly biased prevalence estimates, however, relative differences among survey respondents were to a large degree comparable to relative differences in the total population.

In contrast, survey respondents born outside Sweden differed from persons born outside Sweden in the total population to a degree where they may not be representative and comparisons between this group and other subgroups, using survey data, may be biased.

## Background

Survey data are widely used in social sciences and medical research, but response rates are declining, sometimes to such low levels that the representativeness of the survey responders in relation to the source population and the generalizability of the research results may be questioned [1–3]. However, a low survey response rate does not necessarily bias results per se; only if non-responders differ systematically from the responders on the variables studied.

There are many different reasons for not responding to a survey. Personality characteristics and socio-demographic factors seem to be important; especially educational level has been shown to be consistently associated with willingness to respond to surveys [4]. Individuals interested in the survey topic will respond more readily than individuals with no specific interest [5]. Health surveys are an example of surveys where willingness to respond may be related to the survey topic [6] and where risk of non-response bias must be taken into account.

When studying socioeconomic differences in health and healthcare utilization using survey data, selective non-response may lead to bias, as health status and healthcare utilization are closely related to socioeconomic position [7]. It is however difficult to know in what direction this will bias the results as the health status among the non-responders is unknown. Although most studies of non-responders seem to indicate that non-responders have higher mortality and morbidity than responders [6, 8–12], there are also examples of the opposite [13] as well as examples of studies with no significant differences between respondents and non-respondents [14–16]. When it comes to differences in healthcare utilization among respondents and non-respondents the evidence is limited and not consistent. Some studies suggest that non-respondents have lower healthcare utilization than respondents [13, 17, 18]. However, this concerns primary care or outpatient care [13, 17, 19] and may depend on when in time healthcare data is collected (prior, during or after the survey) [20], the reason for not responding [20] and the type of outcome measure used (cost, number of visits, attended care, etc.).

Whether survey non-response also biases the estimated socioeconomic gradient in health and healthcare utilization is much less investigated and results are inconsistent [11, 21, 22]. In the one study we found which investigated the impact of survey non-response on estimates of socioeconomic differences in healthcare utilization, the results showed that although response bias affected the levels of estimates of healthcare utilization, it did not necessarily affect the estimates of differences between groups [21].

The aim of this study was to compare outpatient healthcare utilization among health survey respondents to that of the total population, and to investigate socioeconomic differences in outpatient healthcare utilization among survey respondents compared to the total population. In many countries the only way of investigating socioeconomic differences in healthcare utilization is by using health surveys as healthcare data is not registered in a way that makes this type of research possible. In the Scandinavian countries and other countries with high quality healthcare registers it is, on the other hand, possible to get this information from registers and healthcare utilization data is seldom collected through surveys. Nevertheless, when studying socioeconomic differences in healthcare utilization, it is important to take need or health status into consideration and therefore survey data on health status may be linked to register data on healthcare utilization for this type of analyses. However, analysing survey respondents instead of the total population may introduce a risk of bias as non-responders and responders may differ significantly in relation to the variables studied.

As all healthcare utilization in Sweden is registered and linked to the individuals using the personal identity number it is possible to determine the healthcare utilization in the total population. We therefore have a unique opportunity to investigate, not just how non-responders differ from responders, but how well survey respondents represent the actual population they are sampled from, concerning healthcare utilization. By linking the information about healthcare utilization to register data on socioeconomic indicators we can investigate socioeconomic differences in healthcare utilization in the total population and compare it to the healthcare utilization of survey respondents.

In this study we used Stockholm County Council’s public health survey from 2010 to investigate whether the survey responders are representative of the total population they are sampled from, regarding outpatient healthcare utilization.

The research questions to be answered in this study are:

- Are responders to health surveys representative of the population they represent regarding outpatient healthcare utilization?
- Do the possible differences influence analyses of socioeconomic differences in outpatient healthcare utilization?

## Method

**Fig. 1:**
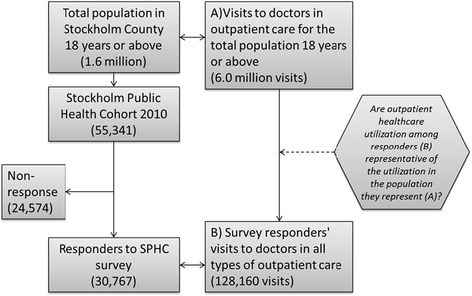
Graphical presentation of the study and the data sources used in the study

We used data on respondents from the Stockholm Public Health Cohort (SPHC), a population-based cohort study commissioned by Stockholm County Council (data and data collection methods is described elsewhere [1]. We use the 2010 sub-cohort with a sampling frame consisting of individuals registered in the total population register who were aged 18 years or above and resident in Stockholm County on the 31^st^ of December 2009, in total 1,601,300 individuals. A sample, stratified on geographical area, of 56,037 persons was drawn from the register. Of these 696 were either deceased or had emigrated and the net sample was 55,341. Of these 30,767 answered the survey corresponding to a response rate of 56 % (Fig. 1).

We know from a previous investigation of the non-response in SPHC that lower income groups and groups with shorter education have lower response rate [23]. The lowest income tertile, had a response rate of 42 % compared to 65 % in the highest income tertile. The group with the lowest educational level had a response rate at 45 % and the group with the highest educational level had a response rate of 63 %. Similar patterns appear between affluent and disadvantaged areas. The highest response rate (64 %) was seen in one of the more affluent areas (Täby) and the lowest response rate (39 %) was seen in one of the most disadvantaged areas (Rinkeby-Kista) [23]. Individuals who had been on sick leave at some point during the year had a lower response rate than individuals not on sick leave (49 vs 56 %) [23].

For the survey respondents we obtained individually linked register data on healthcare utilization in 2010 from Stockholm County Council’s Administrative Database for Analysis and Follow-up of Healthcare Utilization that contains information on all registered outpatient and inpatient care financed by Stockholm County Council. Since universal access to healthcare is a key element in the Swedish welfare model, private health insurance only has a small supplementary function and almost all healthcare is publicly funded by the county councils [24]. The data were anonymized through encrypted personal identity numbers.

Data on socio-demographic background characteristics (age, sex, educational level, income, country of birth and residential area) were obtained from the Longitudinal Integration Database for Social Insurance and Labour Market Studies (LISA) from Statistics Sweden, collecting individually linked variables from different population registers.

For the data on the total population in Stockholm County, from where the SPHC population was sampled, register data from LISA were linked to register data on healthcare consumption, allowing comparison of healthcare utilization among survey respondents to that of the total population, using the same source of register data on healthcare utilization.

### Variables

#### Sex

Sex was determined using the social security number derived from the population register.

#### Age

For the descriptive analyses age was divided into 7 categories (18–29, 30–39, 40–49, 50–59, 60–69, 70–79 and 80+). The age-distribution in the total population, based on these seven categories, was used for the age standardization. In the regression models, where age was controlled for, age was used as a continuous variable.

#### Income

Income was divided into five quintiles, based on annual income, measured as net equalized disposable household income. In income group 1 income was between 1 and 11,644€; in income group 2 between 11,645€ and 16,780€; in income group 3 between 16,781€ and 22,077€; in income group 4 between 22,078€ and 30,934€ and in income group 5 the income level exceeded 30,934€.

#### Education

Education was categorized into 3 different levels: Primary school (9–10 years of schooling or less), Secondary school (at least one year of secondary school) and Post-secondary school (at least one year of post-secondary education).

#### Country of birth

Country of birth was dichotomized into ‘Born in Sweden’ and ‘Born outside Sweden’.

#### Residential area

Residential area was dichotomized into ‘Disadvantaged areas’ and ‘Rest of Stockholm County’. In 1998 disadvantaged residential areas with high levels of unemployment, high proportion of foreign-born residents, low level of education, were identified in the larger Swedish cities for a Metropolitan Development Initiative, which increased resources from state and municipal level during the period 1998–2004 to decrease segregation and improve living conditions. In these areas health is poorer and disease strikes at younger ages [25] and they could therefore be regarded as areas with greater healthcare needs. In this study persons living in a disadvantaged area in Stockholm County were compared to persons living in other areas of the county.

#### Healthcare utilization

Healthcare utilization was measured by the number of visits to doctors in outpatient care (both general practitioners and specialists). Data on healthcare utilisation for both the survey respondents and the total population was derived from the same outpatient care register and consisted of the registered total number of visits from January 1^st^ until December 31^st^ 2010.

### Statistical methods

Data were analysed using SAS 9.4. The mean number of visits and proportion with zero visits among survey respondents was estimated using survey means procedure. In order to compare socio-economic differences in healthcare utilization among survey respondents and in the total population, we used logistic regression analysis for estimating the odds ratio of having at least one visit. Z-tests were done to assess whether the estimates from the survey respondents differed significantly from rates in the general population. As the other outcome variable, the number of visits to outpatient care, is a discrete variable which has a very non-normal distribution, we used negative binomial regression analysis to estimate the relative increase or decrease in the mean number of visits across socioeconomic groups. Among different count data regression models we chose the negative binomial regression model based on goodness of fit measures, reliable estimates, and comparisons of log likelihoods and AIC. We did z-tests to assess whether the estimates from the survey respondents differed significantly from rates in the total population.

## Results

The proportion with at least one outpatient visit to the doctor was higher among survey respondents than in the total population for almost all subgroups, when data were age standardized to the age distribution in the total population. For many subgroups these differences were statistically significant. Among men born outside Sweden 67 % of survey respondents had at least one visit to doctors in outpatient care, compared to 56.5 % among men born outside Sweden in the general population.

The average number of visits among the survey respondents was closer to the average number in the total population, and significantly different only in a few subgroups. Among men the survey respondents tended to have a higher mean number of registered visits than the general population (Table 1).

**Table 1:** Percentage of individuals with at least one visit to a doctor in outpatient care during 2010 in the total population in Stockholm County and the survey population (crude and age standardised)

	Percentage with at least one visit				Mean number of visits			
	Total population	Survey population - age standardised			Total population	Survey population - age standardised		
	%	%	95 % CI (Low)	95 % CI (High)	Mean	Mean	95 % CI (Low)	95 % CI (High)
Women								
Total	74.98	77.03^a^	76.38-	77.69	4.27	4.25	4.16-	4.34
18–29 years^b^	66.37	70.64^a^	68.84-	72.43	2.62	2.76	2.60-	2.91
30–39 years^b^	71.95	74.00^a^	72.38-	75.61	3.33	3.44	3.25-	3.64
40–49 years^b^	70.71	71.56	69.97-	73.15	3.52	3.20^a^	3.02-	3.37
50–59 years^b^	75.15	75.16	73.50-	76.83	4.19	3.95^a^	3.73-	4.17
60–69 years^b^	81.06	83.90^a^	82.60-	85.21	5.05	4.93	4.70-	5.16
70–79 years^b^	88.79	90.04	88.63-	91.45	7.32	7.54	7.14-	7.93
80+ years^b^	92.99	95.06^a^	93.80-	96.31	9.09	9.47	8.96-	9.97
Income quintile 1 (Low)	73.55	76.97^a^	75.58-	78.36	4.22	4.20	4.02-	4.38
Income quintile 2	79.79	79.97	78.76-	81.17	5.17	4.91^a^	4.71-	5.10
Income quintile 3	77.12	78.14	76.64-	79.65	4.33	4.32	4.10-	4.54
Income quintile 4	75.13	74.85	73.23-	76.48	3.73	3.79	3.59-	3.99
Income quintile 5 (High)	72.13	73.01	71.13-	74.89	3.41	3.54	3.33-	3.75
Primary school	82.11	81.50	80.04-	82.96	5.80	5.04^a^	4.81-	5.27
Secondary school	76.99	78.43^a^	77.39-	79.47	4.46	4.66^a^	4.49-	4.82
Post secondary school	72.11	74.06^a^	73.01-	75.12	3.49	3.55	3.43-	3.67
Born in Sweden	76.29	77.16^a^	76.43-	77.89	4.28	4.20	4.10-	4.30
Born outside Sweden	71.08	76.49^a^	74.97-	78.01	4.22	4.46^a^	4.25-	4.67
Non-disadvantaged areas	74.98	77.03^a^	76.35-	77.70	4.25	4.24	4.15-	4.33
Disadvantaged areas	74.99	77.20	73.95-	80.45	4.56	4.48	4.01-	4.94
Men								
Total	61.16	64.05^a^	63.19-	64.92	3.06	3.14	3.05-	3.23
18–29 years^b^	51.12	55.71^a^	53.43-	57.99	1.55	1.75^a^	1.60-	1.89
30–39 years^b^	52.36	54.81^a^	52.69-	56.92	1.76	1.66	1.54-	1.78
40–49 years^b^	56.43	56.85	54.91-	58.80	2.24	2.11	1.96-	2.26
50–59 years^b^	62.94	67.06^a^	65.13-	69.00	3.12	3.37	3.11-	3.63
60–69 years^b^	74.34	77.66^a^	76.12-	79.20	4.67	4.73	4.48-	4.98
70–79 years^b^	85.87	89.00^a^	87.43-	90.57	7.33	7.57	7.08-	8.06
80+ years^b^	92.85	94.18	92.56-	95.79	9.56	9.94	9.24-	10.65
Income quintile 1 (Low)	56.84	61.09^a^	58.78-	63.40	2.74	2.67	2.46-	2.87
Income quintile 2	65.44	67.12	65.07-	69.17	3.66	3.50	3.27-	3.72
Income quintile 3	66.16	68.47	66.46-	70.48	3.59	3.69	3.45-	3.92
Income quintile 4	62.88	62.50^a^	60.76-	64.24	2.92	3.01	2.84-	3.18
Income quintile 5 (High)	60.55	62.90^a^	61.20-	64.59	2.68	2.97^a^	2.79-	3.15
Primary school	68.59	68.61	66.64-	70.58	4.04	3.89	3.66-	4.13
Secondary school	63.54	65.61^a^	64.27-	66.95	3.15	3.20	3.06-	3.34
Post secondary school	57.38	60.58^a^	59.19-	61.98	2.50	2.73^a^	2.60-	2.86
Born in Sweden	62.67	63.44	62.49-	64.39	3.12	3.06	2.96-	3.16
Born outside Sweden	56.50	67.02^a^	65.00-	69.04	2.88	3.51^a^	3.28-	3.73
Non-disadvantaged areas	61.19	63.83^a^	62.94-	64.71	3.06	3.11	3.02-	3.20
Disadvantaged areas	60.71	68.96^a^	64.95-	72.96	3.05	3.64^a^	3.23-	4.06

^a^The estimate is significantly different from true value in the total population

^b^Results of these groups from the survey population were not age-standardised

The most prominent reason for the higher mean number of visits among survey respondents than in the total population was a lower proportion of individuals with zero visits among survey respondents. There were a lower proportion of people with more than five registered visits among survey respondents than among the total population (results are age standardized) (Table 2). Consequently, the mean number of visits among survey respondents, among those with at least one visit, was in most cases lower than in the total population, especially among men (data not shown).

**Table 2:** Distribution of individuals with visits in outpatient care in different categories of visits

	Women		Men	
	Total population	Survey population	Total population	Survey population
	%	%	%	%
1 visit	21.01	22.55	28.33	30.39
2 visits	16.31	17.05	18.17	18.70
3-5 visits	29.63	31.23	27.52	27.82
6-9 visits	17.07	15.91	13.24	12.15
10 or more visits	15.98	13.26	12.75	10.93

We tested a calibration weight designed by statistics Sweden [23] to be used for prevalence estimates. The use of the calibration weight brought the estimates of mean number of visits among survey respondents closer to the mean of the general population, but for most subgroups and for the total survey population the age standardized estimate were closer to the real mean of the general population than the calibrated mean (data not shown), so we chose to continue only with the age standardized estimates.

Tables 3 and 4 shows how relative differences between groups vary between the survey respondents and the total population. In Table 3 the relative odds of having at least one visit in different subgroups compared to their respective reference groups are shown. The z-test shows whether the estimate among survey respondents differs significantly from the total population. For most groups there were no significant differences of the estimate among survey respondents and the total population, but one subgroup stands out. In the total population, individuals born outside Sweden had in comparison with individuals born in Sweden significantly lower odds of having a visit, but among the survey respondents the relationship was opposite (except for older women). Also young men and women in income group 1 among survey respondents had significantly higher odds of having a visit than in the total population, but here the direction of the relationship was the same in both the survey respondents and the total population.

**Table 3:** OR of having at least one visit to a doctor in outpatient care (controlled for age) in the total population and in the survey population

	Total population			Survey population			z-test
	Estimate	95 % CI (Low)	95 % CI (High)	Estimate	95 % CI (Low)	95 % CI (High)	
Women (18–64 years)							
Income quintile 1 (Low)	1.12	1.10-	1.14	1.50^a^	1.32-	1.72	−4.30
Income quintile 2	1.44	1.42-	1.47	1.47	1.29-	1.67	−0.22
Income quintile 3	1.31	1.29-	1.33	1.38	1.21-	1.57	−0.78
Income quintile 4	1.22	1.20-	1.24	1.29	1.14-	1.45	−0.90
Primary school	1.52	1.49-	1.55	1.43	1.26-	1.63	0.86
Secondary school	1.25	1.23-	1.26	1.20	1.10-	1.31	0.95
Born outside Sweden	0.81	0.80-	0.82	1.05^a^	0.94-	1.16	−4.86
Disadvantaged areas	1.10	1.07-	1.13	1.10	0.90-	1.34	0.00
Women (65+ years)							
Income quintile 1 (Low)	0.86	0.82-	0.91	0.94	0.68-	1.30	−0.50
Income quintile 2	1.16	1.11-	1.22	1.30	0.97-	1.75	−0.75
Income quintile 3	1.25	1.19-	1.31	1.53	1.12-	2.09	−1.28
Income quintile 4	1.19	1.13-	1.25	1.25	0.92-	1.70	−0.28
Primary school	0.94	0.90-	0.98	0.96	0.74-	1.24	−0.13
Secondary school	1.01	0.98-	1.05	1.23	0.97-	1.57	−1.57
Born outside Sweden	0.68	0.66-	0.70	0.63	0.50-	0.80	0.57
Disadvantaged areas	0.93	0.86-	1.00	1.16	0.67-	1.99	−0.79
Men (18–64 years)							
Income quintile 1 (Low)	1.20	1.18-	1.22	1.55^a^	1.34-	1.78	−3.44
Income quintile 2	1.46	1.44-	1.48	1.60	1.40-	1.83	−1.34
Income quintile 3	1.36	1.34-	1.39	1.47	1.29-	1.67	−1.15
Income quintile 4	1.19	1.17-	1.20	1.25	1.10-	1.41	−0.78
Primary school	1.58	1.56-	1.61	1.50	1.32-	1.69	0.89
Secondary school	1.31	1.30-	1.33	1.23	1.13-	1.35	1.42
Born outside Sweden	0.82	0.81-	0.83	1.19^a^	1.07-	1.32	−6.82
Disadvantaged areas	1.09	1.06-	1.11	1.32	1.08-	1.61	−1.87
Men (65+ years)							
Income quintile 1 (Low)	0.81	0.76-	0.85	1.11	0.78-	1.59	−1.74
Income quintile 2	1.00	0.96-	1.05	1.17	0.87-	1.59	−0.99
Income quintile 3	1.23	1.18-	1.29	1.44	1.07-	1.94	−1.02
Income quintile 4	1.23	1.17-	1.28	0.88^a^	0.68-	1.14	2.44
Primary school	1.02	0.98-	1.06	1.11	0.86-	1.42	−0.66
Secondary school	1.13	1.09-	1.17	1.14	0.91-	1.43	−0.13
Born outside Sweden	0.74	0.71-	0.77	1.22^a^	0.92-	1.62	−3.44
Disadvantaged areas	0.93	0.86-	1.00	1.45	0.84-	2.51	−1.60

^a^OR in survey population significantly different from the OR in the total population based on the z-test of the two estimates

Reference group: Income quintile 5 (High), Post-secondary school, Born in Sweden, Non-disadvantaged areas in Stockholm county

**Table 4:** Relative difference in average number of visits to doctors in outpatient care (controlled for age) in the total population and in the survey population

	Total population			Survey population			z-test
	Estimate	95 % CI (Low)	95 % CI (High)	Estimate	95 % CI (Low)	95 % CI (High)	
Women (18–64 years)							
Income quintile 1 (Low)	1.42	1.41-	1.43	1.41	1.31-	1.52	0.16
Income quintile 2	1.52	1.50 -	1.54	1.41^a^	1.31-	1.51	2.03
Income quintile 3	1.35	1.34-	1.36	1.34	1.25-	1.44	0.20
Income quintile 4	1.20	1.19-	1.21	1.25	1.17-	1.34	−1.30
Primary school	1.55	1.53-	1.56	1.39^a^	1.30-	1.49	3.02
Secondary school	1.24	1.24-	1.25	1.25	1.19-	1.31	−0.18
Born outside Sweden	1.04	1.03-	1.05	1.12^a^	1.06-	1.18	−2.49
Disadvantaged areas	1.20	1.18-	1.21	1.23	1.10-	1.37	−0.48
Women (65+ years)							
Income quintile 1 (Low)	1.10	1.08-	1.12	1.15	1.03-	1.28	−0.73
Income quintile 2	1.16	1.14-	1.18	1.12	1.02-	1.23	0.67
Income quintile 3	1.20	1.19-	1.22	1.20	1.09-	1.32	0.06
Income quintile 4	1.12	1.10-	1.14	1.10	1.00-	1.22	0.30
Primary school	1.05	1.04-	1.06	0.98	0.90-	1.06	1.75
Secondary school	1.06	1.05-	1.08	1.11	1.03-	1.20	−1.19
Born outside Sweden	1.01	0.99-	1.02	0.98	0.90-	1.06	0.66
Disadvantaged areas	1.05	1.02-	1.08	0.96	0.82-	1.13	1.09
Men (18–64 years)							
Income quintile 1 (Low)	1.59	1.57-	1.61	1.50	1.36-	1.67	1.08
Income quintile 2	1.70	1.68-	1.72	1.58	1.44-	1.74	1.47
Income quintile 3	1.41	1.39-	1.43	1.36	1.24-	1.49	0.82
Income quintile 4	1.18	1.17-	1.19	1.07^a^	0.98-	1.17	2.05
Primary school	1.70	1.68-	1.72	1.61	1.47-	1.75	1.29
Secondary school	1.33	1.32-	1.35	1.19^a^	1.11-	1.27	3.53
Born outside Sweden	0.99	0.98-	1.00	1.20^a^	1.11-	1.29	−4.89
Disadvantaged areas	1.16	1.14-	1.18	1.28	1.11-	1.47	−1.31
Men (65+ years)							
Income quintile 1 (Low)	1.11	1.08-	1.14	1.05	0.91-	1.20	0.84
Income quintile 2	1.15	1.13-	1.17	1.02^a^	0.91-	1.14	2.03
Income quintile 3	1.16	1.14-	1.19	1.03^a^	0.92-	1.14	2.26
Income quintile 4	1.11	1.08-	1.13	0.97^a^	0.87-	1.09	2.27
Primary school	1.07	1.06-	1.09	1.05	0.96-	1.16	0.40
Secondary school	1.06	1.04-	1.07	1.02	0.93-	1.11	0.86
Born outside Sweden	0.99	0.98-	1.01	1.10	0.99-	1.21	−1.90
Disadvantaged areas	0.98	0.95-	1.02	1.13	0.94-	1.35	−1.42

^a^The relative difference in average number of visits in the survey population is significantly different from the relative difference in the total population based on the z-test of the two estimates

Reference group: Income quintile 5 (High), Post-secondary school, Born in Sweden, Non-disadvantaged areas in Stockholm county

Table 4 shows the relative differences in the average number of visits among different subgroups of survey respondents in relation to their respective reference groups in the population and the z-test for the difference between the estimate in the total population and among survey respondents. For some income groups, especially among older men, there were significant differences between the survey respondents and the total population. Again, among men and women aged 18–64 years born outside Sweden, estimates among survey respondents were significantly higher than in the same group in the total population.

## Discussion

According to the results of this study, a greater proportion of respondents to the health survey had been in contact with doctors in outpatient care than the total population. The differences between the survey respondents and the total population were in general greater among men than among women.

The estimates of the mean number of visits among survey respondents were closer to the mean number of registered visits in the total population and significantly different only for a few subgroups. The survey respondents had a higher proportion of people with at least one registered visit, but a lower proportion of people with more than five visits, compared to the total population. When only comparing individuals with at least one registered visit among survey respondents and the total population, the survey respondents actually had fewer visits to outpatient healthcare than the total population.

Whether the differences identified in this study between the survey-respondents and the total population are meaningful will depend on the research question to be addressed. In general there was a greater difference between the survey estimates and the total population concerning the dichotomous variable of having had a visit or not, than concerning the mean number of visits.

Socioeconomic differences in healthcare utilization were similar among the survey respondents and in the total population. For most comparison groups there were no significant differences between estimates of the survey respondents and of the total population. However, among individuals born outside Sweden, in most cases the estimates among survey respondents were not representative of the total population of individuals born outside Sweden. In some cases the estimates from the survey population pointed in the opposite direction of the estimates from the total population for this group. Foreign born individuals usually have lower response rates [26] and the results of this study suggest that foreign born individuals who participate in a health survey may not be representative of foreign born individuals in the population.

Among men aged 65+ years, there was a less steep income gradient in the average number of registered visits among the survey respondents compared to the total population, indicating that basing the estimates only on survey respondents would underestimate the income gradient in the average number of visits in the total population.

That survey respondents had higher utilization of outpatient care than the total population corresponds to earlier studies on the linkage between healthcare utilization and the tendency to be a survey respondent [13, 17, 18]. However, in these previous studies only the risk of having any visit was investigated. In the present study we also analyzed differences in the actual average number of outpatient visits, which closer to the average in the total population. Among those having at least one visit, the survey respondents in many cases had a lower number of registered visits than the total population. Further research is needed to confirm these results in other settings.

The estimation of survey errors due to differential non-response is often done by collecting available register data for the non-response group. This is done in order to assess the potential effects of differential non-response on the results from survey respondents. A strength of this study was the use of population based register data on healthcare utilization, both for the survey respondents and for the total population, which made it possible to directly assess whether the results obtained from respondents to the health survey were systematically deviating from the true value in the total population. This is usually not possible to do and similar studies have had very limited sample size [27]. The use of register based data for both the survey respondents and the total population helped secure the internal validity of the study.

Cross sectional studies are used both to describe the prevalence of different characteristics in a population and to investigate possible associations between variables. Both objectives may be biased by difference in response rates. However, as the results of this study indicate, the second objective might be less affected by response bias, but this is rarely investigated when analysing survey error in relation to selection bias.

This study only analysed outpatient care. The results may have been different if inpatient care had been studied, as it seems that those in greater need of healthcare respond to surveys to a lesser degree, given the fact that fewer individuals with high healthcare utilization participated in this survey.

We were not able to establish when in time healthcare was utilized, in relation to when the survey was completed, which may be seen as a limitation. It has been shown that hospital admission rates for non-responders differ from those of responders depending on whether healthcare data is collected immediately before and during, or after the survey data collection [20]. Although this might be especially important for hospital admission rates, it may also affect utilization of outpatient care.

The results of this study may inform other studies investigating socioeconomic differences in healthcare utilization using health survey data, also in other settings. However, the results may differ in contexts without universal access to healthcare or different healthcare systems. As healthcare utilization may be regarded as a proxy for health status, the results of this study may in some aspects be relevant for researchers using survey data to investigate socioeconomic differences in health in general.

## Conclusion

Compared to the total population, a greater proportion of survey respondents had made at least one outpatient visit to the doctor. However, the mean number of registered visits did not differ significantly between survey respondents and the total population. Hence, depending on the outcome measure used survey-based estimates may result in slightly biased prevalence estimates, however, relative differences among survey respondents were to a large degree comparable to relative differences in the total population.

In contrast, survey respondents born outside Sweden differed from persons born outside Sweden in the total population to a degree where they may not be representative and comparisons between this group and other subgroups, using survey data, may be biased.

## Abbreviations

LISA: Longitudinal Integration Database for Social Insurance and Labour Market Studies
SPHC: Stockholm Public Health Cohort
